# Influence of the initial chemical conditions on the rational design of silica particles

**DOI:** 10.1007/s10971-018-4821-9

**Published:** 2018-09-24

**Authors:** Marion A. Bourebrab, Delphine T Oben, Géraldine G. Durand, Peter G. Taylor, James I. Bruce, Alan R. Bassindale, Alan Taylor

**Affiliations:** 1 National Structural Integrity Research Centre, Granta Park, Great Abington, Cambridge, CB21 6AL UK; 2 School of Engineering, The University of Edinburgh, Edinburgh, EH9 3FB UK; 3 Chemistry Department, The Open University, Walton Hall, Milton Keynes, MK7 6AA UK; 4 TWI Ltd., Great Abington, Granta Park, Cambridge, CB21 6AL UK; 5 Advanced Resins and Coatings Technologies Innovation Centre, School of Engineering, London South Bank University, 103 Borough Road, London, SE1 0AA UK

**Keywords:** Nanoparticles, Silica particles, Sol-gel, Stöber process

## Abstract

The influence of the water content in the initial composition on the size of silica particles produced using the Stöber process is well known. We have shown that there are three morphological regimes defined by compositional boundaries. At low water levels (below stoichiometric ratio of water:tetraethoxysilane), very high surface area and aggregated structures are formed; at high water content (>40 wt%) similar structures are also seen. Between these two boundary conditions, discrete particles are formed whose size are dictated by the water content. Within the compositional regime that enables the classical Stöber silica, the structural evolution shows a more rapid attainment of final particle size than the rate of formation of silica supporting the monomer addition hypothesis. The clearer understanding of the role of the initial composition on the output of this synthesis method will be of considerable use for the establishment of reliable reproducible silica production for future industrial adoption.

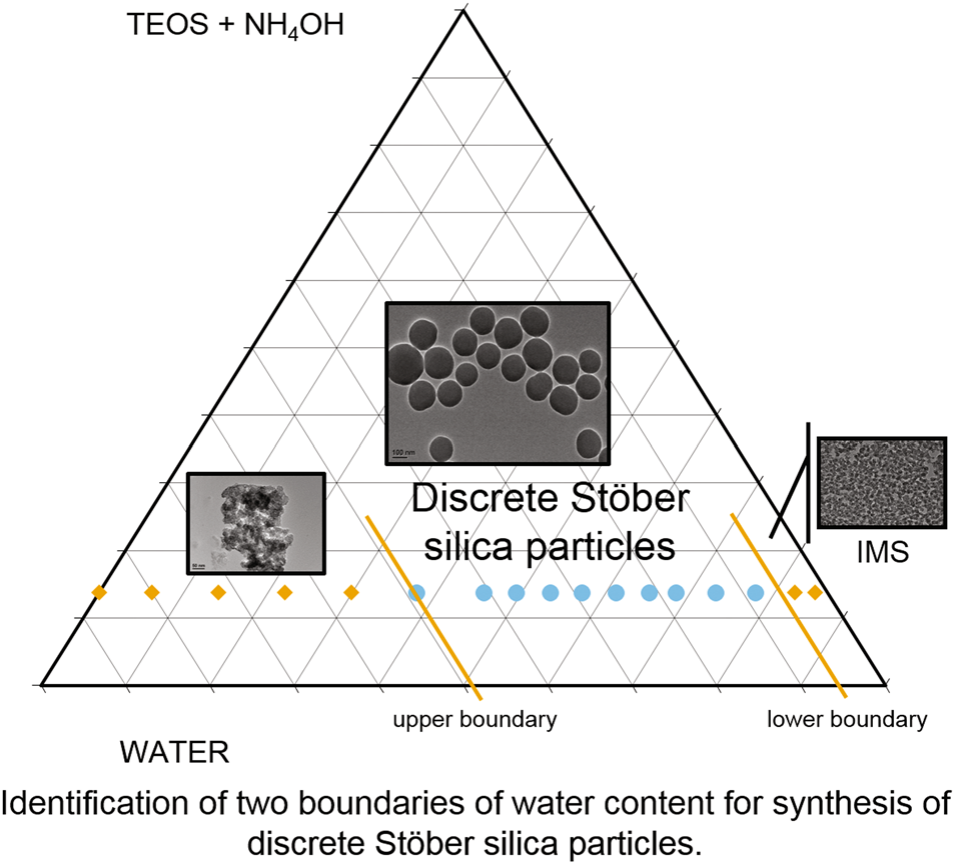

## Highlights

- Identification of compositional regimes for formation of discrete silica particles.
- Influence of composition on morphological characteristics.
- Differentiation between time to completion of silica formation and establishment of particle size.

## Introduction

The use of sol-gel methods allows the fabrication of silica nanoparticles that is alternative to the conventional pyrogenic or precipitation/ion-exchange methods. The potential benefits of this type of fabrication procedure include purer resultant materials [1], reduced energy use in manufacture [2], and, most critically, the potential to control the surface chemistry of the particles [3] and the development of complex structural hierarchies [4, 5]. Stöber, Fink and Bohn [6] are generally accepted as the first reported example of controlled hydrolysis-condensation of alkoxysilanes to produce silica particles in solution. The general reactions are well known and are normally described as follows[7]:

1 $${{{\rm{Si}}\left({{\rm{OC}}_2{\rm{H}}_5} \right)_4}} + x{{{\rm{H}}_2{\rm{O}} \to Si\left({{\rm{OC}}_2{\rm{H}}_5} \right)}}_{4 - x}\left( {{\mathrm{OH}}} \right)_x + x{{{\rm{C}}_2{\rm{H}}_5{\rm{OH}}}}$$

2 $$\equiv {{{\rm{Si}} - {\rm{OC}}_2{\rm{H}}_5 + {\rm{HO}} - {\rm{Si}} \equiv \to \equiv {\rm{Si}} - {\rm{O}} - {\rm{Si}} \equiv + {\rm{C}}_2{\rm{H}}_5{\rm{OH}}}}$$

3 $$\equiv {{{\rm{Si}} - {\rm{OH}} + {\rm{HO}} - {\rm{Si}} \equiv \to \equiv {\rm{Si}} - {\rm{O}} - {\rm{Si}} \equiv + {\rm{H}}_2{\rm{O}}}}$$

Equation 1 corresponds to the hydrolysis reaction, whereas the two others represent the condensation reactions, either liberating alcohol (Equation 2) or water (Equation 3).

The transformation of silicon alkoxides into silica progresses through a series of chemical reactions that have subtle interdependencies. This was described clearly by Assink and Kay [8] who postulated that each of the 15 potential intermediates have slightly different hydrolysis and condensation reaction rates, where the intermediates are described by the general formula Si(OC_2_H_5_)_*x*_(OH)_*y*_(OSi)_*z*_, where *x* + *y* + *z* = 4. The subtleties emerge as a function of the number of hydrolysis reactions that can take place on each silicon-containing moiety; each moiety effectively becomes a new intermediate capable of further reactions. Such reactions include, potentially, further hydrolysis and polymerisation via condensation.

The physical characteristics of the growing polymer also have an obvious, but less well understood, impact on the structural evolution of the silicon oligomer. Brinker and Scherer [9] identified that whilst there were four broad chemical environments for the sol-gel process (high and low water content, high and low pH), three of these resulted in particulates or highly cross-linked oligomers, and only the low water/low pH gave rise to linear polymers with low degrees of cross-linking. The Stöber process is clearly positioned within the high water/high pH environment, where high water is defined as being at least a stoichiometric quantity to hydrolyse all hydrolytically sensitive ligands. If we suppose that all the hydrolysis reactions lead to water liberating condensation reactions and there are no alcohol liberating condensation events, the stoichiometric amount of water needed to transform a tetraethoxysilane into silica is two moles per mole silane (according to Equations 1 and 3). This also assumes all the liberated water is reused to hydrolyse residual silicon-alkoxide bonds. However, if we suppose that liberated water plays a minor, or even negligible, role in secondary hydrolysis (due to hydrolysis being much more rapid than condensation reactions [10]), then the effective stoichiometric amount of water required would be one mole of water per mole of hydrolysable groups at the beginning of the reaction, so for a tetraalkoxysilane a molar ratio of 4:1 would be required.

The basic synthesis routine behind the formation of silica Stöber spheres is relatively straightforward, the silane source (typically tetraethoxysilane, TEOS) is introduced to a water and alcohol mixture, in which a basic catalyst such as ammonia is added. Several research groups have analysed a number of synthesis parameters including reagents concentrations and temperature, and their impact on particle size [11–13]. Process variables in the synthesis methodology have also been examined in detail. Jafarzadeh et al. [14] and Guo et al. [15] investigated the influence of feed rates and addition sequences on the resultant silica nanoparticles, with smaller particles being generated under lower rate of ammonia addition. Park et al. [16] established that the temperature of reaction had a greater influence on the final particle size than the concentration of reagents and the feeding rate, and gave their optimal conditions to synthesise 13 nm particles.

Considerable debate exists surrounding the mechanism of particle formation and growth [2, 17]. Bogush et al. [11] concluded that growth is shelf-sharpening: as particle growth occurred, the size distribution became narrower. They also stated that there was a single mechanism behind nucleation and growth, but that the emergent nuclei were unstable and aggregated. Ibrahim et al. [7] determined that if the synthesis conditions were such that the rate of hydrolysis was increased, this would lead to fewer but larger particles. Green et al. [18] established that there were two stages to growth: initial nucleation via monomer addition and later growth due to aggregation. This theory is also supported by Okudera and Hozumi [19]. Topuz et al. [20] favoured a diffusive type growth model, and demonstrated a clear induction period for the growth of the particles after mixing of the reagents.

**Fig. 1:**
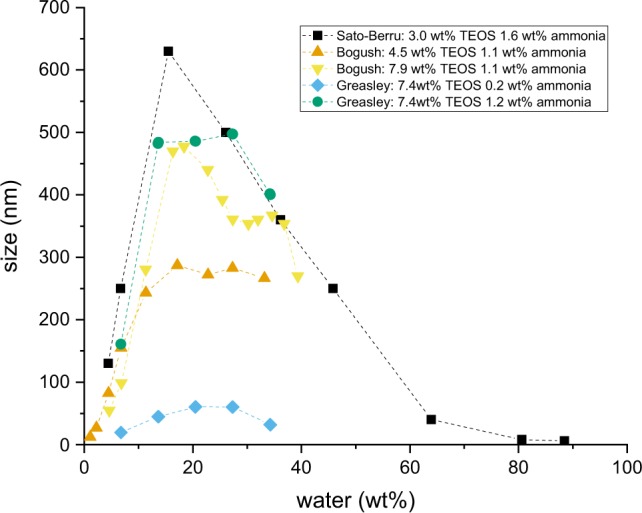
Particle size relation to water content, as reported by Sato-Berru et al. [22], Bogush et al. [11] and Greasley et al. [33]. The data was replotted in wt% of water for this graph

Whilst the detailed mechanisms of nucleation and growth are still to be elucidated, a clear understanding of the influence of composition and processing parameters on the resultant particle size and its distribution is slowly emerging. Overall, it has been demonstrated that the particle size increased with increasing water concentration to a maximum, and then decreased as the water content is further increased, for different concentrations of TEOS and ammonia, as is shown in Fig. 1. It is interesting to note that generally the biggest particles were synthesised with a water content comprised between 10 and 30 wt%, and that after 35 wt% of water, the size decreased. This would mean that past this specific initial concentration of water in the system, bigger particles cannot be stabilised in their medium anymore. Bridger et al. [21] mentioned the existence of a saturation limit of water at 15 mol/L of water (corresponding to 34 wt% of water), in a Stöber system containing methanol as solvent. With increments of water, the particles would rise to a peak size, and their size would decline past this saturation concentration. Although the reason for this maximum is unclear from the literature and is yet to be explained, it is interesting to note that researchers have mainly focussed on systems which water contents represented only half or less of the whole systems. In the example of Sato-Berru et al. [22], they went beyond 60 wt% of water, but overlooked the miscibility gap that occurred at those higher water contents.

In general, it is difficult to precisely compare the studies reported by different authors since the initial synthesis parameters vary considerably. However, given the potential importance of this synthesis method as the basis for the development of complex structural hierarchies, such as raspberry structures, a clearer understanding of compositional boundaries and the underpinning design rules is required. Our aim is to identify key compositional regimes that have a significant impact on the formation of Stöber spheres from tetraethoxysilane under conditions of high pH; a secondary objective is to determine within these compositional regimes the influence, particularly of water content, on the resultant silica particles.

## Experimental

### Materials

In this study, all the silica particles were synthesised via the Stöber process [6], at room temperature. Tetraethoxysilane (TEOS) was used as the silica precursor (Silanes & Silicones, Stockport, UK). Ammonium hydroxide (Sigma-Aldrich, Gillingham, UK) was diluted in-house from 28–30 wt% to 25 wt%. Industrial methylated spirit (IMS) (99% ethanol, 1% methanol), supplied by VWR International (Lutterworth, UK) was used as the co-solvent. Finally, water was deionised in-house using a Purelab Prima pump (Elga LabWater, High Wycombe, UK) operating to deliver water which hardness is below 20 ppm.

### Silica particle synthesis

Seventeen formulations containing TEOS, IMS, ammonia and water were prepared. The total weight of the formulations was the same (weighing 636 ± 1 g), and the quantities of TEOS and ammonia were nominally identical for each formulation. Within the formulation series, the total water content increased from formulation 1 to formulation 17. To ensure each formulation had the same final weight, the quantity of ethanol was reduced as the water content increased. The quantities by weight percentage of the reagents are shown in Table 1. Each solution was prepared following a two-pot process. The TEOS was mixed thoroughly with 28% of the IMS to be used in a given formulation. This was then added rapidly to a mixture of the water, ammonia, and the remaining of IMS. The solutions were then shaken for 2 min to homogenise and left to react at room temperature.

**Table 1:** Relative quantities (in weight percentage (wt%)) of the reagents for the Stöber synthesis of silica

Formulation #	TEOS	IMS	Ammonia	Water
1	13.19	84.89	0.48	1.44
2	13.21	82.52	0.47	3.79
3	13.24	77.76	0.47	8.53
4	13.21	73.12	0.48	13.20
5	13.25	68.35	0.48	17.91
6	13.24	65.15	0.48	21.14
7	13.21	61.25	0.48	25.06
8	13.18	57.26	0.47	29.09
9	13.20	53.46	0.47	32.86
10	13.23	49.48	0.48	36.81
11	13.20	45.57	0.48	40.75
12	13.23	37.64	0.48	48.65
13	13.19	29.92	0.47	56.42
14	13.22	22.05	0.48	64.26
15	13.18	14.23	0.48	72.11
16	13.23	6.32	0.48	79.98
17	13.25	0.05	0.48	86.23

The stoichiometric amount of water to enable complete hydrolysis of the four ligands of TEOS for these systems is four moles of water per mole of TEOS used, if we choose not to take into account the water liberated during the reaction. By using the same amount of TEOS in each formulation, this 4:1 molar ratio equates to having a formulation which water content equals 4.56 wt%. This value is just above that of formulation 2. The 2:1 molar ratio between water and TEOS corresponds to 2.28 wt% of water, which is greater that of formulation 1. This means that the synthesis according to formulation 1 has a significantly substoichiometric water content for silica to be synthesised. Particles from formulation 2 are also not fully hydrolysed (below the 4:1 ratio), but silica formation could occur (above the 2:1 ratio). The reactions for formulations 3 and beyond are not limited by the availability of reagents to enable hydrolysis.

For the purposes of comparison, a second set of formulations were prepared, the details of these are given in Table 2. Ethanol rather than IMS was used to remove the potential complication of alcohol exchange, slightly higher TEOS and ammonia concentrations were also used. These modifications were adopted to provide a second set of samples that followed the broad formulation methodology but with different initial compositional details. Such differences are typical of slightly different approaches reported in the literature as previously discussed; this second set of materials was prepared specifically to provide samples for TEM analysis.

**Table 2:** Relative quantities (in weight percentage (wt%)) of the reagents for the second set of formulations for TEM analysis

Formulation #	TEOS	Ethanol	Ammonia	Water
2b	19.65	74.30	0.57	5.48
3b	19.65	70.52	0.57	9.26
5b	19.65	62.96	0.57	16.82
7b	19.65	55.40	0.57	24.38
8b	19.66	51.61	0.57	28.17
9b	19.65	47.85	0.57	31.93
11b	19.66	40.26	0.57	39.51
12b	19.66	32.70	0.57	47.07
14b	18.94	20.58	0.55	59.93
16b	19.66	2.46	0.57	77.32

### Characterisation methods

#### Particle size measurements

The particle diameters were determined by the Dynamic Light Scattering (DLS) method. The Zetasizer Nano-S (Malvern Instruments, UK) is equipped with a laser beam of *λ* = 632.8 nm and a backscatter detector positioned at 173^◦^. Each sample was measured from the parent solution without any modification, the particle size distribution was recorded at least three times for each formulation analysed, as well as at different times during the reaction process (between 0.22 and 48 h) to track the particle growth. All measurements were performed at room temperature (25 ± 0.1 °C), with the attenuation set to automatic. The reported value of Z-average corresponds to the measurement of the hydrodynamic diameter of the particles [23, 24], also named the cumulants mean, which is the most stable parameter produced by the DLS method. The polydispersity index (PdI) was also measured. A PdI value smaller than 0.1 indicates a monodisperse distribution of particles [25].

#### Imaging via transmission electron microscopy (TEM)

The silica dispersions were dried under vacuum for 24 h. Each sample was then imaged via TEM JEM2100 (JEOL, USA), which is equipped with a lanthanum hexaboride crystal emitter at 200 kV, giving a point resolution of 0.25 nm. For the analysis, the dried materials were deposited on a carbon grid. The resulting images were processed with the ImageJ software.

#### Non-volatile and solid contents

The non-volatile content (NVC) was measured gravimetrically by drying an aliquot of each silica dispersion for 18 h at 65 °C and applying the calculation method shown in Equation 4. This was done 48 h after mixing when the hydrolysis reactions are presumed to have progressed to completion. In addition, the progression of the reactions leading to the silica particle synthesis was evaluated by calculating the NVC of each dispersion at various times of reaction, during 80 h. This was done to determine whether any volatile silica species (most likely unreacted TEOS) were present in the mixture.

4 $${\mathrm{NVC}}\% = \frac{{{\mathrm{weight}}\,{\mathrm{of}}\,{\mathrm{dried}}\,{\mathrm{silica}}\,{\mathrm{particles}}}}{{{\mathrm{weight}}\,{\mathrm{of}}\,{\mathrm{dispersion}}\,{\mathrm{sampled}}}} \times 100$$

In addition, aliquots of each formulation were dried for 72 h at 65 °C, in order to be characterised via thermogravimetric analysis (TGA, Netzsch STA 449 F3 Jupiter equipped with silicon carbide furnace, Netzsch, Germany) to evaluate the final solid content. These analyses were carried out in a Pt-Rh crucible, from room temperature to 1000 °C at a rate of 10 °C/min, in air.

#### Surface area and porosity

Gas adsorption and desorption at the gas/solid interface allows the assessment of a wide range of pore sizes, including the complete range of micro- and mesopores. The Brunauer-Emmett-Teller (BET) theory [26] was applied to calculate the surface area of each sample, within the BET range (0.05 6 *P/P*_0_ 6 0.3). The mesoporosity of each sample was analysed by the application of a modified Kelvin equation, the Barrett-Joyner-Halenda (BJH) method [27]. A different approach to calculate the effective pore size distributions from nitrogen adsorption isotherms in microporous materials was adopted. The Horvath–Kawazoe method (HK) [28, 29] is a semi-empirical and analytical method which relies on a slit-pore shape geometry of materials. An extension of the HK method was followed here, the Saito-Foley model [29–31], which uses a cylindrical pore geometry approach. The experiment was carried out with a Micromeritics ASAP 2060 (Micromeritics Instrument Corporation, Norcross, GA, USA) and the results were analysed with the software Micromeritics MicroActive^TM^. A sample of each formulation of silica particles was dried for 18 h at 65 °C. Each sample weighed between 0.10 and 0.15 g. They were then degassed under vacuum, at 90 °C for 1 h, followed by 16 h at 150 °C. This protocol avoided damaging the samples [32]. Nitrogen (N_2_) was selected as the adsorption/desorption gas for the BET analysis. The experiments were carried out at −196 °C (77 K) by immersing the flask containing the sample in a dewar filled with liquid nitrogen.

## Results

### Overview

**Fig. 2:**
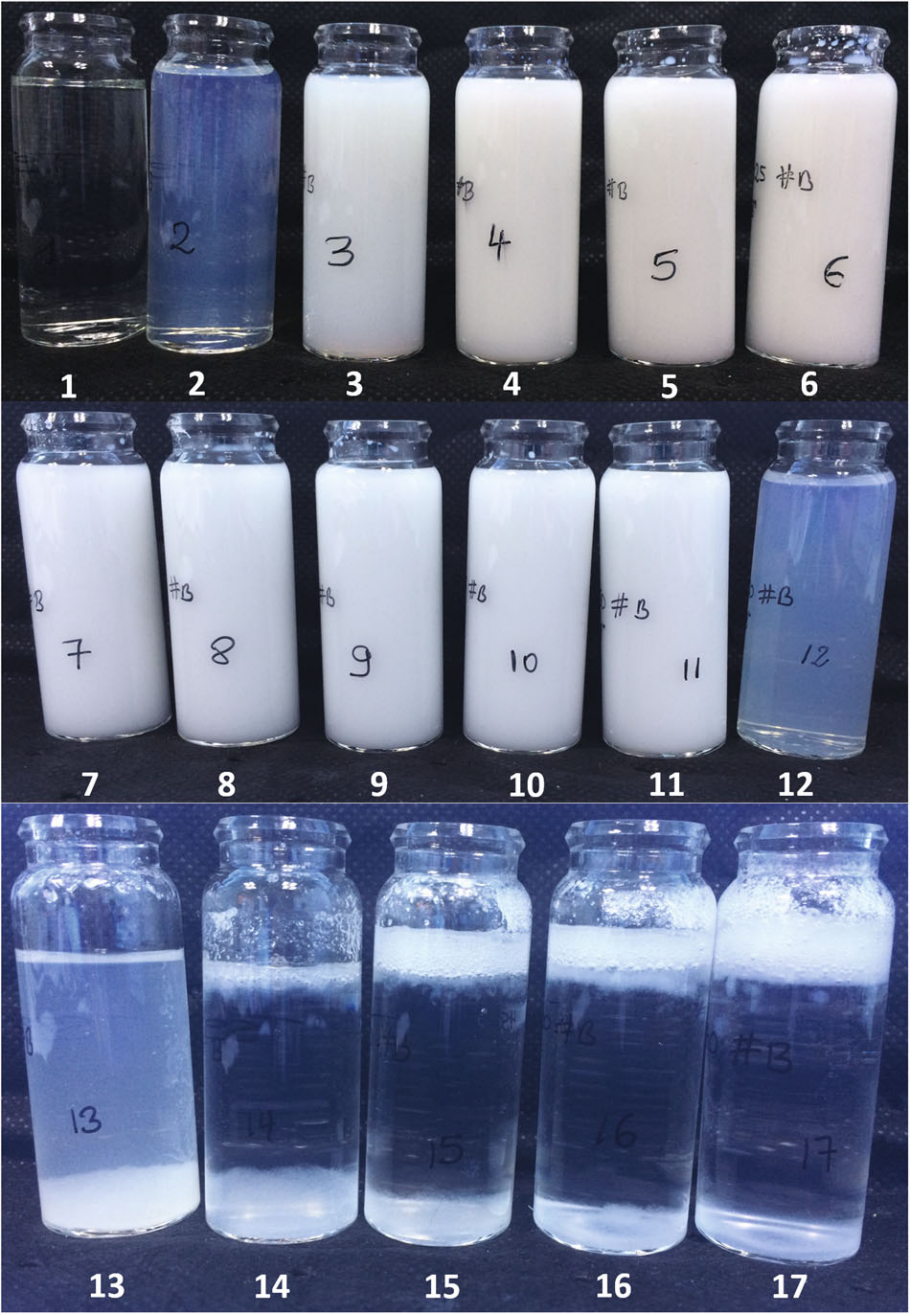
Visual assessment of the 17 formulations after 48 h of reaction

The prepared suspensions were characterised according to the methods described in Section 2.3, the results of these various analyses are presented in Table 3. A visual assessment was undertaken at different times after mixing the reagents, Fig. 2 shows a photograph of the suspensions after 48 h of reaction. The first formulation was transparent; the second one presented a clear single phase mixture that had a slight blue haze typical of nanoparticles (<100 nm) in suspension. Formulations 3–11 where opaque single phase suspensions with no sign of sedimentation. Formulation 12 was a single phase liquid but was clear with a blue haze similar to formulation 2. Formulations 13–18 where multiphase mixtures with some sedimentation, a transparent/translucent phase and a third phase which had a foam-like appearance floating on the surface.

**Table 3:** Summary of the characteristics of the prepared suspensions and their dried solids

Formulation #	Size (Z-average, nm)	Size (as measured by TEM, nm)	PdI	BET surface area (m^2^/g)	Total pore volume (cm^3^/g) at 0.99 P/P_0_	Isotherm hysteresis type [35]	Residual weight TGA (%)	NVC %
1	12.50		0.35	549.92	0.51	H2	71.93	4.26
2	32.01		0.20	347.31	0.41	H2	85.37	4.31
2b		15.58						
3	122.50		0.06	233.69	0.34	H1	87.50	4.32
3b		68.88						
4	234.35		0.05	165.08	0.32	H1	86.94	4.30
5	351.75		0.06	103.78	0.18	H4	88.23	4.30
5b		169.12						
6	579.20		0.15	197.58	0.18	H4	85.17	4.23
7	727.05		0.05	220.37	0.15	H4	88.59	4.28
7b		358.91						
8	754.00		0.09	283.74	0.19	H4	90.89	4.15
8b		625.66						
9	818.95		0.07	295.03	0.19	H4	88.73	4.25
9b		656.79						
10	676.70		0.05	307.86	0.21	H4	88.52	4.17
11	451.15		0.02	290.54	0.35	H4	86.02	4.17
11b		482.81						
12	198.13		0.41	251.74	0.67	H2	91.05	4.12
12b		82.79						
13	*		*	444.92	0.60	H2	90.70	4.11
14	*		*	453.09	0.59	H2	91.02	4.12
14b		16.46						
15	*		*	468.15	0.60	H2	91.82	3.92
16	*		*	447.85	0.57	H2	92.22	3.95
16b		9.94						
17	*		*	464.71	0.55	H2	92.22	3.44

*Those values could not be measured by the DLS method due to the two-phase nature of the systems

### Dependence of size with water content

**Fig. 3:**
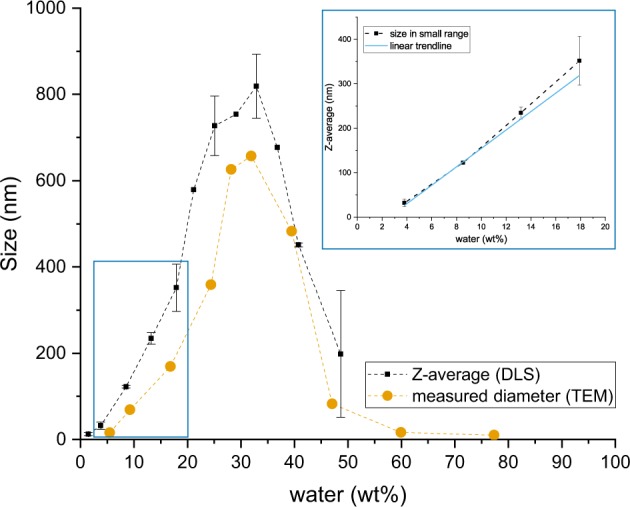
Dependence of particle size with water content. The error bars represent the standard deviation of several measurements at a same water content. The insert highlights the linear increase of size in the lower range of water content (between 3.79 and 17.91 wt%). The trendline has a correlation coefficient of r^2^ = 0.984

**Fig. 4:**
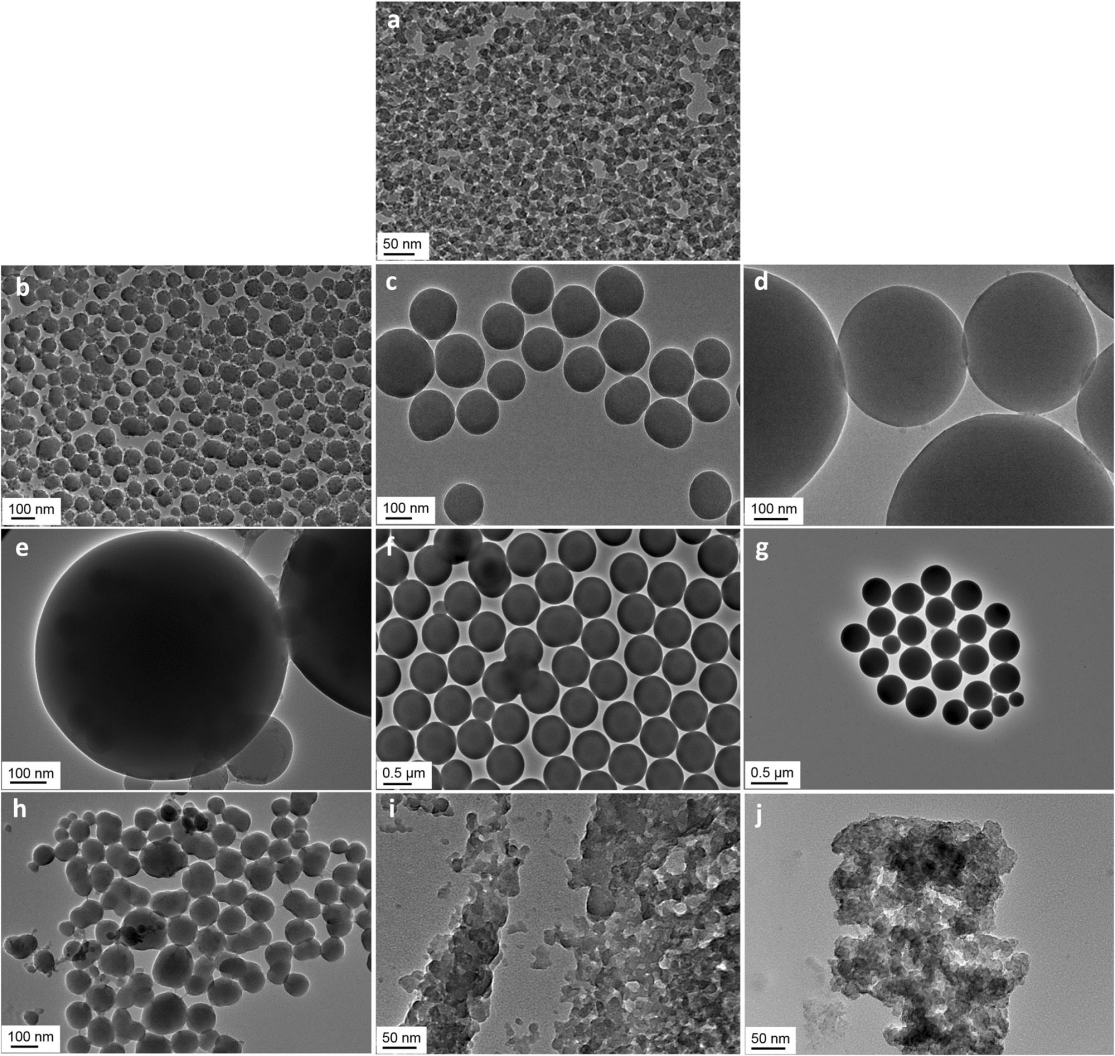
TEM images of dried silica made from formulations with increasing water contents. **a** formulation 2b; **b** formulation 3b; **c** formulation 5b; **d** formulation 7b; **e** formulation 8b; **f** formulation 9b; **g** formulation 11b; **h** formulation 12b; **i** formulation 14b; and **j** formulation 16b. Courtesy of The Open University

Increasing the water content of the silica dispersion has a strong influence on the size of synthesised particles [11, 22, 33]. As shown in Fig. 3, for each and above showed as multiple phase systems, with the silica being present as aggregated secondary structures rather than discrete, individual particulates, which is visible in the TEM images corresponding to formulations 12b to 16b in Fig. 4h–j.

The fact that there is a particle size peak that occurred at around 33 wt% of water, and is reported by other research groups, indicates that there might be a change of stability of the siloxane intermediates or the nascent particles at this water content. It is well known that silicon alkoxides such as TEOS are incompatible with water and phase separate when mixed, which is why alcohols are used as co-solvents. At low water content, the mixture is homogeneous with respect to TEOS and water molecules, therefore the reaction and structural pathways are likely to follow a relatively narrow distribution. At higher water content, this homogeneity is not present initially, and is only reached as alcohol is liberated during hydrolysis (Equation 1) at the water/TEOS interface. Whilst the initial mixture has its water content increased from the condensation reaction (Equation 3), the amount of liberated alcohol needed to achieve homogeneity increases until a point at which a single phase cannot be achieved. Given the high pH of these mixtures, condensation is likely to be rapid following hydrolysis. The structural evolution in a homogeneous mixture is therefore likely to be very different to that of a two-phase emulsion. This will be the case even if the emulsion is temporary, as the liberated ethanol stabilises a single phase mixture. This can considered from a compositional perspective using the nomenclature of Assink and Kay [8] Si(OC_2_H_5_)_*x*_(OH)_*y*_(OSi)_*z*_. Specifically, TEOS (*x* = 4; *y* = *z* = 0) is insoluble in water, and in water/alcohol mixtures above a critical water content. Close to this critical value, the silane composition changes to *x* 6 = 4 and *y* > 0, followed by *z* > 0. The 13 intermediate species between TEOS and SiO_2_ will have different solubility characteristics. Knowing that the composition of the medium changes since alcohol is liberated during hydrolysis mainly, and water is then liberated during the condensation reaction. The solubility of the evolving silane/siloxanes would impact whether migration could occur across a phase boundary: the availability of (partially) hydrolysed silanes to participate in condensation reactions would then be dependent on local compositional considerations. Viewed in these terms, it is reasonable to think that the homogeneity of the initial mixture would dictate the structural characteristics and uniformity of the resultant particles. In addition, between 3.79 and 17.91 wt% of water, which corresponds to formulations 2 to 5 (see Table 1), i.e. the systems with lower quantities of water (excluding formulation 1), the size increased linearly with the quantity of water, as is illustrated in the insert in Fig. 3. This observation concurs with results of Van Helden et al. [34] with particles of diameters ranging from 20 to 300 nm. Knowing the linear relationship between the two parameters means that any size of particles in the range 30 to 400 nm could be easily predicted and synthesised accordingly.

### Induction period

**Fig. 5:**
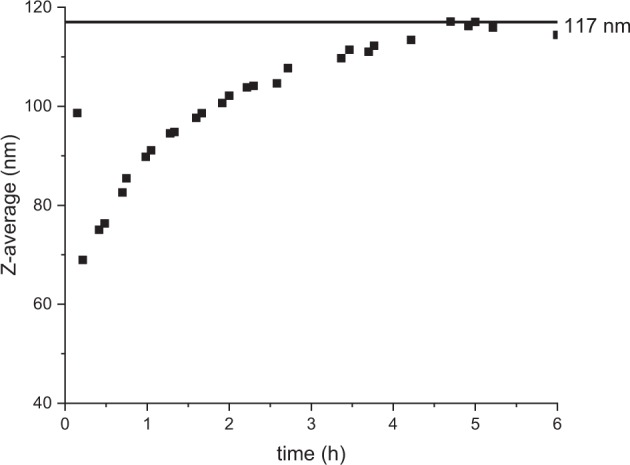
Induction period for formulation 3, with the Z-average reaching a plateau at 117 nm after 5 h of reaction

In order to better understand the mechanisms in place during particle growth, the Z-average of the dispersions were measured over time, from 0.22 to 5 h after initial mixing of the reagents. In a review, Hyde et al. [2] established that particles synthesised via the Stöber process could grow resulting from two mechanisms, or a combination of both: either small nuclei aggregate to form a bigger particle (aggregation model), or small nuclei grow over time (nucleation growth model). We reported here the example of formulation 3, for which the particle size distribution during the induction period showed a pattern that could be attributed to the nucleation growth model. The nuclei grew from 69 nm at 0.22 h to 117 nm after 5 h, at which value the average diameter plateaued, as can seen from Fig. 5. This feature was verified by Topuz et al. [20], but for particles of greater diameters (310 and 520 nm).The similarity for different sizes would mean that the model of nucleation growth

increment of water, the silica particles increased in size, peaking at ~33 wt% of water, and thereafter their size reduced. Imaging dried particles via TEM confirmed the change in size, which is illustrated in Fig. 4. This imaging method demonstrates the clear spherical appearance of the silica particles synthesised with formulation 3b to 11b (Fig. 4b, g). The images corresponding to formulations 2b, 12b, 14b, and 16b (Fig. 4a, h–j), however, show agglomerates. In Section 2.2, we calculated that the quantity of water needed for full hydrolysis was 4.56 wt%, above that of formulation 2. Knowing that formulation 2b uses a slightly greater content of TEOS, this threshold of full hydrolysis corresponds to 6.79 wt% of water, which is also greater than that of formulation 2b. Therefore, the materials synthesised via formulations 1, 2, and 2b were particles not yet fully formed (we can see on the TEM image in Fig. 4a that the dried material was close to be of spherical shape, yet still presented agglomeration). Formulations 12 is valid across a range of size of particles.

**Fig. 6:**
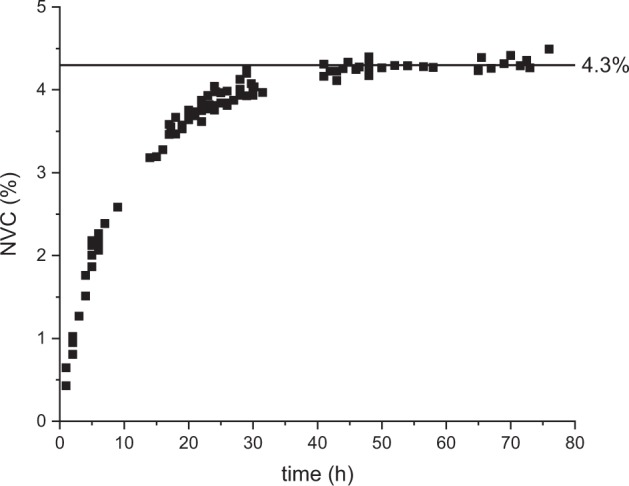
Evolution of non-volatile content of formulation 2 over time. The NVC plateaued at 4.3% after 40 h of reaction

Nevertheless, another parameter that should be taken into account to determine whether the nucleation growth has terminated is the availability of the volatile precursor (TEOS). Measuring the NVC throughout the synthesis gives a good estimate of the total time required for the TEOS to be converted into non-volatile species. Complete reaction from TEOS to silica would result in a final NVC value of 3.8% (based on the initial quantity of TEOS). However, obtaining a higher value suggests that not all condensation reactions occurred, and some oligomers still contained some ethoxy and silanols entities (*x* and *y* greater than zero in Assink and Kay’s formulation [8]). In fact, the NVC value was maintained between 4.2 and 4.3% until formulation 11, after which it decreased, which confirms the change of reaction products, towards species with negligible *y* values and greater *z*. In Fig. 6, the example of NVC monitoring for formulation 2 is displayed. The NVC plateaued at 4.3% after 40 h of reaction, which means that hydrolysis and subsequent condensation reactions were still ongoing even after the particle size was established.

After the induction period, which was 5 h from the onset of reaction, the particle size distribution was measured at 6, 22, 30, and 48 h. Once past the induction period, the average diameter remained overall constant over time for the formulations measured via the DLS method. The final size for each formulation was overall reached after 6 h, and is indicated in Table 3. DLS measurements could only be taken for formulations 1–12. The higher water content formulations did not allow size estimation by DLS due to the multiple phase nature of the systems. TEM analysis showed that formulations with a higher water content than 56 wt% were highly aggregated and agglomerated structures instead of discrete particles.

### Porosity and surface area

**Fig. 7:**
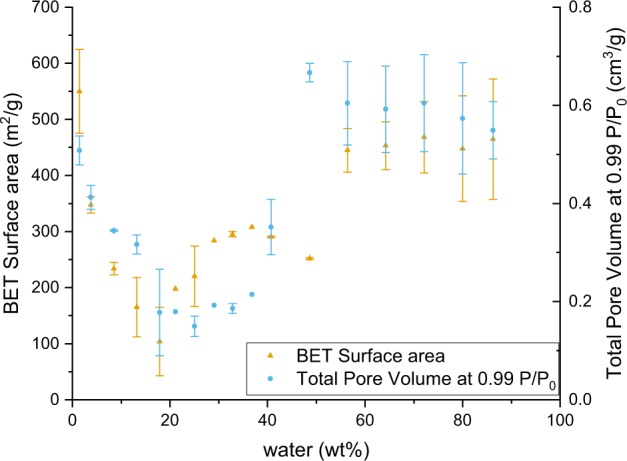
Surface area measurements via the BET method (measured in the BET range), and total pore volume (measured at 0.99 P/P_0_). The error bars correspond to the standard deviation between all measurements done per formulation

The surface area and micro- and mesoporosity of all samples were analysed using nitrogen adsorption/desorption methods. All the samples showed type IV isotherms according to the IUPAC classification [35] (typical of mesoporous materials with a uniform surface). The results of the measured surface area and total pore volume are shown in Table 3 and Fig. 7. The BET data also suggests that the materials prepared using greater than stoichiometric amounts of water could be grouped in two separate categories:

- formulations 3–11, with an average surface area of ~230 m^2^/g and an average total pore volume of 0.23 cm^3^/g;
- formulations 13–17 which were made with water contents greater than 56 wt%, and which had an average surface area of ~450 m^2^/g and an average total pore volume of 0.58 cm^3^/g.

**Fig. 8:**
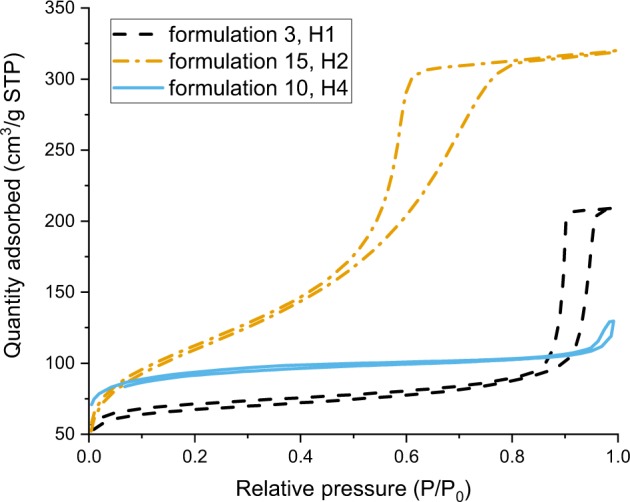
Isotherms with the three hysteresis curves: H1, H2, and H4, and their corresponding formulations

The first category, formulations 3–11 demonstrated lower surface areas and pore volumes. Within this category, two behaviours were observed: the hysteresis curve for particles made from formulations 3 and 4 was in the H1 class (according to the IUPAC classification [35]), meaning that uniform spheres with a narrow pore size distribution were synthesised. The hysteresis curved for the materials made from formulations 5–11 were representative of the narrow slit micropores of the materials (type H4). The second category of materials (formulations 13–17), had higher total pore volumes and surface areas, and the hysteresis curves were characteristic of disordered materials with a less well defined pore shape (type H2). Figure 8 shows the three representative examples of hysteresis types we observed. The particles

synthesised with formulation 12 seemed to be an intermediate between the two categories of materials: the estimated surface area suggests this material should belong to the first category, whilst the measured total pore volume and the shape of the hysteresis curve are similar to those of the second category.

Finally, with regards to formulations 1 and 2, it was clear from the TEM image for the latter formulation (Fig. 4a), that particles were not completely formed but agglomerates were instead. We calculated in Section 2.2 that the system made from formulation 2 did not contain enough water to be fully hydrolysed, therefore formation of spherical silica nuclei was altered.

### Thermogravimetric analysis

**Fig. 9:**
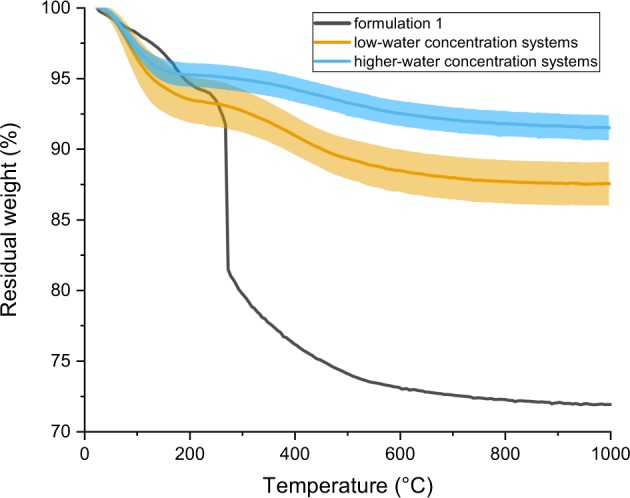
Thermogravimetric analysis of the 17 formulations, showing the difference between the first category of formulations (2–11 here), with a lower residual weight than the second category (12–17 here); formulation 1 is represented on its own. The shaded areas around the curves represent the standard deviation within each category of formulations

The residual weight of the samples on heating to 1000 °C are given in Fig. 9. The data is presented in a consolidated manner for the materials produced from formulations 2–11, and for 12–17. The behaviour of the sample from formulation 1 was clearly different and so is presented separately. Considering the weight loss behaviour of sample 1, the initial weight loss is likely to be due to physisorbed species, which would be removed by evaporation on heating. The significant weight loss at about 270 °C can be explained by considering the initial formulation. The substoichiometric level of water used in the preparation of this materials would mean that the TEOS could not be fully hydrolysed and so the resultant material would not be just silica, or hydroxylated silica, but would have residual chemically bound ethoxy groups. Thus considering the resultant material in terms of Assink and Kay’s nomenclature

*Si*(*OC*_2_*H*_5_)_*x*_(*OH*)_*y*_(*OSi*)_*z*_ [8], *x*, *y* and *z* would all be different from zero. The consolidated thermogravimetric curves of the remaining materials exhibited a similar pattern of behaviour. A relatively low temperature weight loss (up to ~200 °C) which is most likely to be due to loss of water and physisorbed volatiles remaining after the initial drying of the samples [36]. The second weight loss stage, between 200 °C and 700 °C, is most likely to be caused by dehydroxylation (removal of silanol groups) [37–39].

Within these broad descriptions, the key differences between the two categories of materials were related to the absolute weight loss over the entire temperature range studied. The materials produced using water contents above 48 wt% showed lower levels of weight losses in the low and higher temperature ranges. On average, samples made from formulations 12–17 lost ~4.8% of weight on heating to 200 °C and had a final residual weight of 91.5%. Samples made from formulations 2–11 lost an average 6.5% on heating to 200 °C, and had an average final residual weight of 87.5% on heating to 1000 °C. This behaviour appears contradictory to the nitrogen sorbtion data since the samples made from formulations 12–17 have a greater specific surface area than those made from formulations 2–11. However, these samples 2–11 show a greater weight loss due to dehydroxylation. This indicates that the materials synthesised from formulations 2–11 are richer in silanols than samples 12–17, therefore present a higher silanol density. This also suggests that the two categories of samples (2–11 and 12–17) exhibit different silanols populations. Other potential explanations include higher residual alkoxy content in samples 2–11; however, the lack of notable weight loss in the 300–400 °C region (as seen for the substoichiometric formulation 1) would mitigate against this.

## Discussion

The Stöber method has been applied across a wide range of compositions. The primary reactants used were tetraethoxysilane, methanol denatured ethanol, deionised water and ammonia. The resultant materials have been examined and analysed using a number of characterisation methods. In-line with the findings of other researchers, we have found a variation in particle size as a function of water content of the initial mixture. It is clear from our findings and those of others that water content in the synthesis step is a key process parameter, and perhaps the critical factor, in determining the characteristics of the resultant material.

We postulate that the resultant materials can be categorised from the perspective of the initial chemical composition if a rapid mixing strategy, common in Stöber-like methodologies, is adopted. Moreover, the categorisation appears to be dictated by the initial water content. The rapid hydrolysis under base catalysis required a stoichiometric ratio of four moles of water per mole of TEOS. This ratio appeared to represent a threshold value, which we describe as a lower boundary condition for true Stöber sphere synthesis. Lower quantities of water led to materials with high porosity, high surface area and considerable residual organic content as a result of incomplete hydrolysis. Using the notation of Assink and Kay [8], *x*, *y* and *z* were all non-zero and so the transition towards silica was incomplete. As the initial water content increased above the stoichiometric level, the resultant materials appeared to have common characteristics with regards to total pore volume and residual weight. The particle size increased with increasing water content, as was demonstrated in Section 3.2 when particle sizes were measured using light scattering or direct measurement using TEM methods. This observation was valid even when minor modifications were made such as changes in ammonia level or the grade of ethanol. Examination by TEM indicated discrete and uniform particles at higher water contents than the lower boundary. As the initial water content increased, a transition between 40 wt% and 48 wt% of water occurred, where the particles were no longer uniform and discrete but showed signs of aggregation. Other characteristics also changed after this compositional range that we define as the upper boundary. The BET surface area of the dried particles appeared to markedly increase, as did the total pore volume, and typical nitrogen adsorption/desorption isotherm changed to reflect a less defined pore distribution of a disordered material.

Thermogravimetry analysis of the materials produced between the upper and lower boundary compositions (corresponding to the low water concentrations systems in Fig. 9) and those produced at higher initial water content indicated a subtle but interesting difference. The higher initial water content materials appeared to lose less weight during dehydroxylation even though they exhibited greater surface areas. This would be consistent with a scenario where there would be a difference in the type of silanol present on the surface of the material dependent on whether the samples were produced from a composition above or below the upper boundary. For example, if the discrete particles produced between the boundary conditions were rich in geminal silanols (and so had a higher *Q*^2^/*Q*^3^ ratio), whilst the aggregated particles were rich in isolated or vicinal silanols (and had a lower *Q*^2^/*Q*^3^ ratio), the observed behaviour would be explained. This forms a testable hypothesis that could be used to investigate our compositional postulate.

The visual appearance of the suspensions also clearly supports the idea of compositional regimes within the range of formulations we have studied. Formulation 1 was far below the stoichiometric level and appeared as a clear, colourless liquid. Formulation 2 was fractionally substoichiometric and yielded a typical, small particle containing suspension showing Rayleigh scattering. As the initial water content increased above stoichiometric levels, the increase in particle size simply yielded single phase white/opaque suspensions demonstrating Mie scattering. Formulation 11 appeared to be the final one in this series and so marks the last sample before the upper boundary condition. This correlates well with the TEM images. As the initial water content increased, the visual appearance changed significantly and compositional homogeneity was lost as two or more phases were formed.

The use of alcohols such as ethanol in sol-gel systems is well established and widely accepted due to the need to achieve chemical compatibility, as they act as co-solvent between water and the hydrophobic alkoxides. In our opinion, the upper boundary limit is likely to be due to issues of solubility, either initially of the TEOS or of some of the resultant intermediates of proceeding hydrolysis and condensation. The fact that the upper boundary limit does not correlate with the particle size maxima does not discount the possibility that they are linked. The initial composition may not be homogeneous, but this may rapidly change during the mixing procedure due to two linked effects. Firstly, water will be consumed by reaction with the TEOS and secondly ethanol will be simultaneously liberated as a by-product of this hydrolysis. The net effect would be to make the composition richer in alcohol, which would increase the chemical compatibility and reduce the tendency to phase separation. In particular, the use of co-solvents other than IMS would provide insight into the role of solvent characteristics on particle behaviour and resultant size. Again, this is a testable hypothesis, which, whilst outside the scope of this study, could provide valuable insight into the mechanisms driving the behaviour we have observed.

## Conclusion

**Fig. 10:**
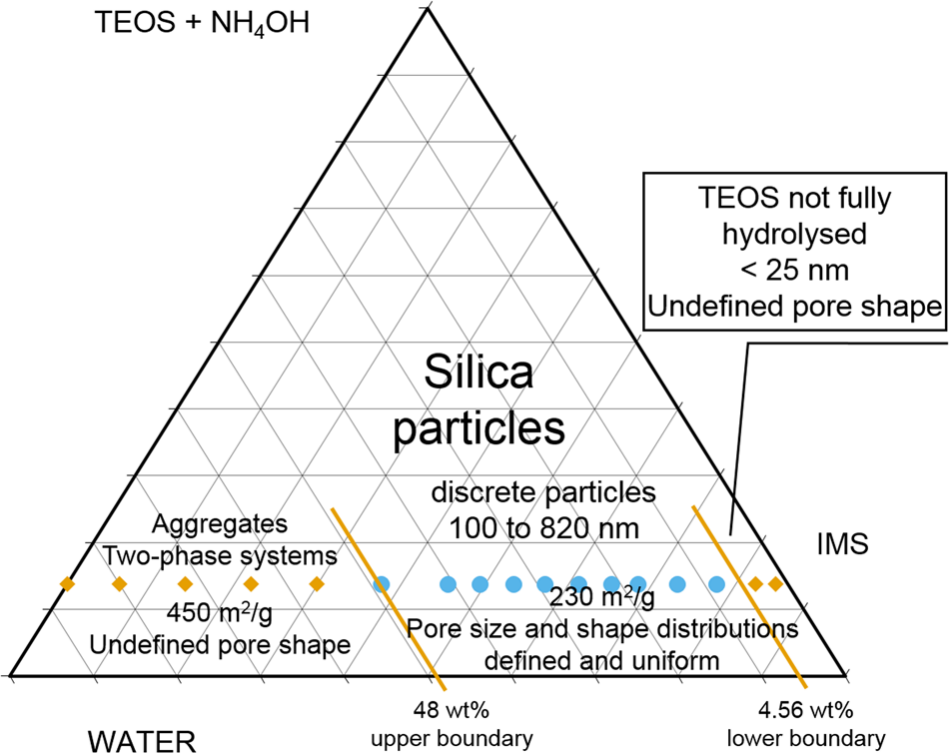
Ternary diagram highlighting the threshold values of water between which silica particles can be synthesised for the selected quantities of the other reagents used in this study

It is clear that a complex series of chemical behaviours clearly dictate the structural evolution of TEOS-derived silicas. As with most sol-gel synthesis routines the practical undertaking appears straightforward, but the resultant materials are dependent on a raft of process parameters. However, the compositional approach we have adopted does allow some design guidelines to be elucidated. Specifically, we postulate a lower boundary condition at the stoichiometric amount of water. We also postulate an upper boundary condition that exists between 40 wt% and 48 wt% of water, but whose influence may appear at even lower water content. Between these two boundary conditions, uniform and discrete particles are formed where particle size can be controlled by the initial water content. The pore structure of these particles appears consistent, as does the dehydroxylation characteristics. These boundary conditions provide compositional guidance to the reproducible synthesis of these particles, and can be represented as a ternary compositional diagram as we propose in Fig. 10. Clearly, our compositions form a linear tie-line which is why the diagram is limited at present. However, we believe this approach will be of value in terms of providing a framework by which Stöber silica materials can be compared across different formulation methodologies leading to a deeper and more comprehensive understanding of the chemical and structural events that are taking place.

## References

[1] Lim HM, Lee J, Jeong JH, Oh SG, Lee SH (2010). Engineering.

[2] Hyde ED, Seyfaee A, Neville F, Moreno-Atanasio R (2016). Ind Eng Chem Res.

[3] Rahman Ismail Ab, Padavettan Vejayakumaran (2012). Synthesis of Silica Nanoparticles by Sol-Gel: Size-Dependent Properties, Surface Modification, and Applications in Silica-Polymer Nanocomposites—A Review. Journal of Nanomaterials.

[4] Perro A, Reculusa S, Bourgeat-Lami E, Duguet E, Ravaine S (2006). Colloid Surf A.

[5] Tsai HJ, Lee YL (2007). Langmuir.

[6] Stöber W, Fink A, Bohn E (1968). J Colloid Interface Sci.

[7] Ibrahim IAM, Zikry AAF, Sharaf MA (2010). J Am Sci.

[8] Assink RA, Kay BD,(1984) ^1^H NMR Studies of the Sol Gel Transition. *MRS Proceedings* **32** 301

[9] Brinker CJ, Scherer GW (1989). Sol-gel Science: the Physics and Chemistry of Sol-gel Processing.

[10] Matsoukas T, Gulari E (1988). J Colloid Interface Sci.

[11] Bogush G, Tracy M, Zukoski C (1988). J Non-Cryst Solids.

[12] Rao KS, El-Hami K, Kodaki T, Matsushige K, Makino K (2005). J Colloid Interface Sci.

[13] Qi D, Lin C, Zhao H, Liu H, Lü T (2017). J Dispers Sci and Technology.

[14] Jafarzadeh M, Rahman IA, Sipaut CS (2009). J Sol-Gel Sci andTechnology.

[15] Guo Q, Yang G, Huang D, Cao W, Ge L, Li L (2018). Colloid Polym.

[16] Park SK, Kim KD, Kim HT (2002). Colloids Surf A.

[17] Han Y, Lu Z, Teng Z, Liang J, Guo Z, Wang D, Han MY, Yang W (2017). Langmuir.

[18] Green DL, Lin JS, Lam YF, Hu MZC, Schaefer DW, Harris MT (2003). J Colloid Interface Sci.

[19] Okudera H, Hozumi A (2003). Thin Solid Films.

[20] Topuz Berna, Şimşek Deniz, Çiftçioğlu Muhsin (2015). Preparation of monodisperse silica spheres and determination of their densification behaviour. Ceramics International.

[21] Bridger K, Fairhurst D, Vincent B (1979). J Colloid Interface.

[22] Sato-Berrú R, Saniger JM, Flores-Flores J, Sanchez-Espíndola M (2013) J Mater Sci Eng A 3(4):237

[23] International Organization for Standardization. BS ISO 22412:2008: Particle size analysis - Dynamic light scattering (DLS) (2008)

[24] Thomas JC (1987). J Colloid Interface Sci.

[25] Stetefeld J, McKenna SA, Patel TR (2016). Biophys Rev.

[26] Brunauer S, Emmett PH, Teller E (1938). J Am Chem Soc.

[27] Barrett EP, Joyner LG, Halenda PP (1951). J Am Chem Soc.

[28] Horvath G, Kawazoe K (1983). J Chem Eng Jpn.

[29] Haghighatju F, Hashemipour Rafsanjani H, Esmaeilzadeh F (2017). Micro; Nano Lett.

[30] Saito A, Foley HC (1991). AIChE J.

[31] Lowell S, Shields JE, Thomas MA, Thommes M (2004) *Characterization of Porous Solids and Powders: Surface Area, Pore Size and Density*. Springer, Dordrecht, The Netherlands. 10.1007/978-14020-2303-3

[32] Li S, Wan Q, Qin Z, Fu Y, Gu Y (2015). Langmuir.

[33] Greasley SL, Page SJ, Sirovica S, Chen S, Martin RA, Riveiro A, Hanna JV, Porter AE, Jones JR (2016). J Colloid Interface Sci.

[34] Van Helden AK, Jansen JW, Vrij A (1981). J Colloid Interface.

[35] Sing K, Everett D, Haul R, Moscou L, Pierotti R, Rouquerol J, Siemieniewska T (1985). Pure Appl Chem.

[36] Dzis’ko V, Vishnevskaya A, Chesalova V (1950). Russ J Phys.

[37] Armistead CG, Hockey JA (1967). Trans Faraday.

[38] Effati E, Pourabbas B (2012). Powder Technol.

[39] Zhuravlev LT (2000) Colloids Surfaces A 173:1–38

